# Blood Pressure, Readmission, and Mortality Among Patients Hospitalized With Acute Kidney Injury

**DOI:** 10.1001/jamanetworkopen.2024.10824

**Published:** 2024-05-13

**Authors:** Benjamin R. Griffin, Mary Vaughan-Sarrazin, Qianyi Shi, Patrick Ten Eyck, Heather S. Reisinger, Korey Kennelty, Mary K. Good, Melissa L. Swee, Masaaki Yamada, Brian C. Lund, Diana I. Jalal

**Affiliations:** 1 Center for Access Delivery & Research and Evaluation (CADRE) Center, Iowa City VA Health Care System, Iowa City, Iowa; 2 Department of Medicine, University of Iowa Carver College of Medicine, Iowa City; 3 The University of Iowa College of Pharmacy, Iowa City

## Abstract

**Importance:**

Acute kidney injury (AKI) complicates 20% to 25% of hospital admissions and is associated with long-term mortality, especially from cardiovascular disease. Lower systolic blood pressure (SBP) following AKI may be associated with lower mortality, but potentially at the cost of higher short-term complications.

**Objective:**

To determine associations of SBP with mortality and hospital readmissions following AKI, and to determine whether time from discharge affects these associations.

**Design, Setting, and Participants:**

This retrospective cohort study of adults with AKI during a hospitalization in Veteran Healthcare Association (VHA) hospitals was conducted between January 2013 and December 2018. Patients with 1 year or less of data within the VA system prior to admission, severe or end-stage liver disease, stage 4 or 5 chronic kidney disease, end-stage kidney disease, metastatic cancer, and no blood pressure values within 30 days of discharge were excluded. Data analysis was conducted from May 2022 to February 2024.

**Exposure:**

SBP was treated as time-dependent (categorized as <120 mm Hg, 120-129 mm Hg, 130-139 mm Hg, 140-149 mm Hg, 150-159 mm Hg, and ≥160 mm Hg [comparator]). Time spent in each SBP category was accumulated over time and represented in 30-day increments.

**Main Outcomes and Measures:**

Primary outcomes were time to mortality and time to all-cause hospital readmission. Cox proportional hazards regression was adjusted for demographics, comorbidities, and laboratory values. To evaluate associations over time, hazard ratios (HRs) were calculated at 60 days, 90 days, 120 days, 180 days, 270 days, and 365 days from discharge.

**Results:**

Of 237 409 admissions with AKI, 80 960 (57 242 aged 65 years or older [70.7%]; 77 965 male [96.3%] and 2995 female [3.7%]) were included. The cohort had high rates of diabetes (16 060 patients [20.0%]), congestive heart failure (22 516 patients [28.1%]), and chronic lung disease (27 682 patients [34.2%]), and 1-year mortality was 15.9% (12 876 patients). Overall, patients with SBP between 130 and 139 mm Hg had the most favorable risk level for mortality and readmission. There were clear, time-dependent mediations on associations in all groups. Compared with patients with SBP of 160 mm Hg or greater, the risk of mortality for patients with SBP between 130 and 139 mm Hg decreased between 60 days (adjusted HR, 1.20; 99% CI, 1.00-1.44) and 365 days (adjusted HR, 0.58; 99% CI, 0.45-0.76). SBP less than 120 mm Hg was associated with increased risk of mortality at all time points.

**Conclusions and Relevance:**

In this retrospective cohort study of post-AKI patients, there were important time-dependent mediations of the association of blood pressure with mortality and readmission. These findings may inform timing of post-AKI blood pressure treatment.

## Key Points

**Question:**

Following an episode of acute kidney injury (AKI), what is the ideal blood pressure range, and how soon after hospital discharge do potential benefits of lower blood pressure outweigh risks of mortality and readmission?

**Findings:**

In this cohort study of 80 960 patients from the Veterans Healthcare Association who had AKI during a hospital admission, systolic blood pressure of 130 to 139 mm Hg had the most favorable postdischarge risk level for mortality and readmission over time. There was a clear, time-dependent mediation of the associations of blood pressure with mortality and readmission, with patients with higher blood pressures generally being at lower risk for mortality and readmission initially, but at higher risk later in the postdischarge course.

**Meaning:**

Among patients post-AKI, there were significant, time-dependent mediations on the associations of blood pressure with mortality and readmission, which may inform the ideal degree and timing of post-AKI blood pressure treatment.

## Teaser

This cohort study analyzes the association of blood pressure with readmission and mortality over time among patients hospitalized with acute kidney injury (AKI) in the Veterans Health Administration system.

## Introduction

Acute kidney injury (AKI) complicates 20% to 25% of all hospital admissions^1^ and is associated with significant increases in postdischarge morbidity and mortality.^2^ In fact, over one-quarter of all patients discharged following an AKI event will die within the following year,^3,4^ with cardiovascular disease (CVD) being the leading cause.^4^ Furthermore, post-AKI patients in the Veteran’s Health Administration (VHA) system have a higher prevalence of existing CVD and cardiovascular risk factors than the general population, including congestive heart failure (CHF), complicated hypertension, diabetes, chronic kidney disease (CKD), proteinuria, and peripheral vascular disease.^5^

Blood pressure reduction is critical for both primary and secondary prevention of major cardiovascular disease events, heart failure, and cardiovascular mortality,^6,7,8^ and as a result, major guidelines recommend that all adults with high CVD risk should be treated to achieve a blood pressure target of less than 130 mm Hg systolic blood pressure (SBP) and 80 mm Hg diastolic blood pressure.^9^ However, despite the increased risk of CVD and death in individuals after AKI, optimal blood pressure targets and optimal timing of blood pressure control for this group remain undefined.

While the higher rates of CVD and CVD-related mortality in the post-AKI population suggest a likely benefit from blood pressure reduction, there are concerns that early, intensive blood pressure lowering might result in harms including rehospitalization due to recurrent AKI or electrolyte disturbances such as hyperkalemia. These concerns are supported by the findings of several observational studies in adults without AKI,^10,11,12^ suggesting that aggressive blood pressure control in the inpatient setting, as well as escalation of blood pressure control upon discharge, are associated with increased rates of short-term adverse events, including AKI.

Studies in the post-AKI population have generally focused on outcomes associated with use of angiotensin converting enzyme inhibitor (ACEI) or angiotensin receptor blocker (ARB) medications but have not often examined blood pressure.^13^ These studies suggest that ACEI or ARB use is associated with long-term reductions in mortality,^14^ but potentially at the cost of increased short-term rehospitalization, AKI, and hyperkalemia.^13,14,15^ Although the findings of short-term harm associated with ACEI or ARB use have not been universal,^16,17^ nearly one-half of patients taking blood pressure medications, particularly ACEI or ARB medications, are not restarted on these agents following an AKI event.^18,19^

A major evidence gap therefore remains regarding the association of blood pressure with post-AKI outcomes, and the degree to which time from discharge is associated with risks and benefits. Our main objectives in this analysis were to determine the associations of SBP with mortality and hospital readmission, and to determine whether time from hospital discharge affects these associations in the post-AKI population. Based on existing literature, we hypothesized that lower SBP would be associated with higher rates of short-term complications but lower rates of long-term mortality.

## Methods

This cohort study was approved by the institutional review boards and Research and Development Committee at the Iowa City VA Health Care System as part of a larger study with previous publications.^20,21,22^ A waiver of informed consent was granted for this retrospective study because the study used deidentified data in accordance with the Common Rule. The study followed the Strengthening the Reporting of Observational Studies in Epidemiology (STROBE) reporting guidelines.

### Data Source

We obtained data from the VA Informatics and Computing Infrastructure. We identified admissions in VA hospitals and retrieved laboratory, vital sign, and mortality data in the Corporate Data Warehouse inpatient files.

### Study Population

**Figure 1.**
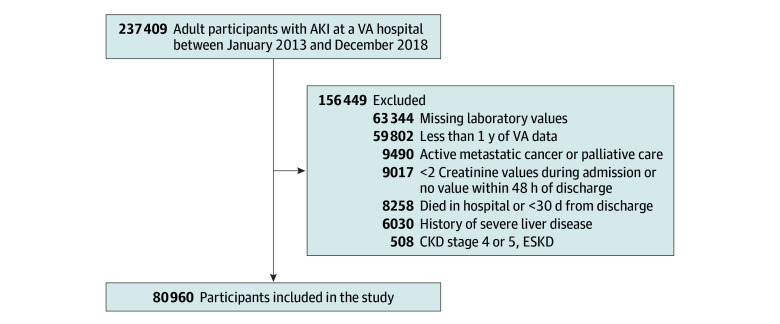
Patient Flow Diagram AKI indicates acute kidney injury; CKD, chronic kidney disease; ESKD, end-stage kidney disease; VA, Department of Veterans Affairs.

We included all adult patients older than 18 years of age within the VHA system from January 1, 2013, to December 31, 2018, with an acute hospital admission complicated by AKI, defined using the Kidney Disease Improving Global Outcomes (KDIGO) guidelines^23^ as an increase in creatinine of 0.3 mg/dL (to convert to micromoles per liter, multiply by 88.4) or greater within 48 hours or greater than 50% from baseline within 7 days. Baseline creatinine was the median creatinine value from 6 months to 7 days prior to the index admission. Eligible patients were required to have at least 1 year of laboratory data within the VA system prior to the index admission, and at least 1 creatinine value prior to the index admission. Multiple admissions from the same patient were included if the admissions with AKI occurred more than 1 year apart. Patients were excluded if (1) creatinine or proteinuria data were not available from the admission; (2) the patient had severe or end-stage liver disease, stage 4 or 5 CKD, end-stage kidney disease, or metastatic cancer; (3) the patient died during the index hospitalization or within 30 days of hospital discharge; or (4) the patient did not have at least 1 blood pressure measurement within 30 days after discharge (Figure 1). CKD stages 4 and 5 were excluded because of lack of data from trials like the Systolic Blood Pressure Intervention (SPRINT) trial,^8^ and their exclusion from KDIGO blood pressure guidelines. Patient race and ethnicity were identified from the VHA Corporate Data Warehouse, and categorized as American Indian or Alaska Native, Asian, Black or African American, Hispanic or Latino, Native Hawaiian or Other Pacific Islander, White, or unknown. Race and ethnicity were included to account for possible disparities in blood pressure control following AKI.

### Exposure Variable and Outcomes

SBPs were treated as time-dependent (categorized as <120 mm Hg, 120-129 mm Hg, 130-139 mm Hg, 140-149 mm Hg, 150-159 mm Hg, and ≥160 mm Hg). Time spent in each SBP category was accumulated over time and represented in 30-day increments. The primary outcomes were time to mortality following discharge and time to all-cause rehospitalization at a VA hospital.

### Adjustment Variables

Covariates in the model were age; comorbidities including chronic lung disease, cancer, unexplained weight loss, dementia, CHF, and tobacco use; admission laboratory values including hematocrit, blood urea nitrogen, bilirubin, and albumin; and kidney variables including AKI stage, baseline creatinine, discharge creatinine, and degree of proteinuria during hospitalization. Comorbid conditions were defined using previously published algorithms^24^ based on diagnoses on inpatient and outpatient claims incurred during the 12 months prior to admission. Proteinuria was categorized as none (albumin-creatinine ratio [ACR] <30 mg/g or negative dipstick protein), mild (ACR 30-300 mg/g or dipstick protein trace –1^+^), or severe (ACR >300 mg/g or dipstick protein ≥2^+^). The remaining laboratory values were categorized using cutoffs defined in the Acute Physiology, Age, Chronic Health Evaluation-III algorithm.^25^

### Statistical Analysis

Means and SDs or counts and percentages were used to describe the distributions of continuous and categorical variables, respectively. We used discrete-time survival models to evaluate the relative hazard of death in monthly intervals up to 12 months after discharge, allowing time spent within each SBP category to accumulate over sequential months. Models included interactions between time since discharge and SBP category to accommodate nonproportional relative hazards. We calculated hazard ratios (HRs) for each SBP category relative to SBP greater than or equal to 160 mm Hg at 6 different time points over the follow-up period (60 days, 90 days, 120 days, 180 days, 270 days, and 365 days after hospital discharge). Models used robust standard errors with an exchangeable working correlation matrix to account for the multiple observations per patient. Patients alive after 12 months were censored. For the secondary outcome of all-cause hospital readmission, we also censored for mortality. To address the potential for a type I error due to multiple comparisons, Bonferroni correction was used, and therefore a *P* value < .01 was considered significant and 99% CIs are presented.

To graphically illustrate changes over time, monthly projected mortality or all-cause readmission was calculated for the average patient, for whom all other adjustment variables were set to mean values. We also used unadjusted data to create graphs of absolute mortality and absolute readmission at each time point, allowing patient groups to vary based on the most recent SBP category. All analyses were performed using SAS software version 9.4 (SAS Institute). Data analysis was conducted from May 2022 to February 2024.

#### Secondary Analysis: ACEI and ARB

Previous studies have found associations of ACEI and ARB use with improved long-term mortality in the post-AKI population, but also with higher short-term rates of recurrent AKI and hyperkalemia.^13,14,15^ To evaluate ACEI and ARB outcomes in this cohort, we conducted an additional analysis to compare outcomes by SBP over time in patients who were and were not prescribed ACEI or ARB within 60 days of discharge. Models included a 3-way interaction term between time since discharge, cumulative SBP category, and ACEI or ARB use. Interaction terms were used to calculate the hazard of death and readmission for each blood pressure category relative to SBP of 160 mm Hg or greater at 60 days, 180 days, and 365 days from hospital discharge, and to determine relative risks for mortality and readmission in those with and without ACEI or ARB use for each blood pressure category and time point.

#### CHF Sensitivity Analyses

The post-AKI population has a large number of patients with CHF who may be more sensitive to lower blood pressures than the general post-AKI population.^26^ As a sensitivity analysis, we repeated the previously described modeling after excluding patients with CHF at the time of the index admission.

#### AKI Stage Sensitivity Analyses

Severe AKI is often used as a criterion for closer follow-up or specialty referral after discharge.^27^ We also conducted a stratified analysis in those with a maximum of stage 1 AKI compared with patients with a maximum of stage 2 or 3 AKI, including those who received renal replacement therapy while admitted.

## Results

### Population Characteristics

There were 80 960 eligible patient admissions (57 242 aged 65 years or older [70.7%]; 77 965 male [96.3%] and 2995 female [3.7%]; 18 436 Black or African American [22.8%]; 5606 Hispanic or Latino [6.8%]; 52 757 White, [65.2%]) (Figure 1 and Table 1). The number of patients with multiple AKI admissions occurring over a year apart was negligible (14 cases). The cohort had high rates of comorbidities including hypertension (68 980 patients [86.0%]), CHF (22 516 patients [28.1%]), chronic lung disease (27 682 patients [34.2%]), and diabetes (16 060 patients [20.0%]), and mortality at 1 year occurred in 12 876 patients (15.9%). Patients typically had stage 1 AKI during their admission (63 588 patients [78.5%]). ACEI or ARB medications were prescribed within 60 days of discharge to 35 098 patients (43.8%). Patient characteristics based on the first blood pressure following discharge are shown in eTable 1 in Supplement 1. The group with SBP less than 120 mm Hg had the highest rates of CHF (8383 of 24 074 patients [34.8%]) and chronic lung disease (9184 of 24 074 patients [38.1%]). Patients with SBP of 160 mm Hg or greater had the highest proportion of Black or African American patients (1377 of 3263 patients [28.2%]) and the highest mean (SD) baseline creatinine values (1.7 [1.1] mg/dL vs 1.3 [0.8] mg/dL for the overall cohort). Age and admission laboratory values were otherwise similar among groups.

**Table 1.** Cohort Characteristics

Characteristics	Participants, No. (%) (N = 80 960)
Age, y	
<45	1662 (2.4)
45-59	10 910 (13.5)
60-64	11 146 (13.8)
65-69	18 873 (23.3)
70-74	14 523 (18.0)
75-84	15 323 (18.9)
>84	8523 (10.5)
Sex	
Male	77 965 (96.3)
Female	2995 (3.7)
Race and ethnicity	
American Indian or Alaska Native	556 (0.7)
Asian	293 (0.4)
Black or African American	18 436 (22.8)
Hispanic or Latino	5506 (6.8)
Native Hawaiian or Other Pacific Islander	537 (0.7)
White	52 757 (65.2)
Unknown	2875 (3.6)
Hypertension	68 980 (86.0)
Initial postdischarge SBP, mm Hg	
<120	23 755 (29.6)
120-129	19 825 (24.7)
130-129	18 549 (23.1)
140-149	9968 (12.4)
150-159	4881 (6.1)
≥160	3239 (4.0)
ACEI or ARB use within 60 d of discharge	35 098 (43.8)
Diabetes	16 060 (20.0)
Unexplained weight loss	5188 (6.5)
Dementia	4040 (5.0)
Congestive heart failure	22 516 (28.1)
Chronic lung disease	27 682 (34.2)
Tobacco use	17 311 (21.4)
Baseline serum creatinine, mean (SD), mg/dL	1.3 (0.8)
Discharge serum creatinine, mean (SD), mg/dL	1.5 (1.4)
Kidney replacement therapy during admission	936 (1.2)
Kidney recovery at discharge^a^	44 519 (55.2)
Kidney recovery at 90 d^a^	63 881 (79.2)
Acute kidney injury category	
Stage 1	63 588 (78.5)
Stage 2	7955 (9.8)
Stage 3	9417 (11.6)
Proteinuria (baseline, 1 y before admission)	
Normal	54 799 (67.7)
Mild	22 890 (28.3)
Heavy	3271 (4.1)
Proteinuria (most abnormal during admission)	
Normal	24 097 (29.8)
Mild	38 448 (47.5)
Heavy	11 659 (14.4)
Missing	6756 (8.4)
Albumin, g/dL	
≤1.9	1729 (2.1)
2.0-2.4	5409 (6.7)
2.5-4.4	69 962 (86.4)
≥ 4.5	3850 (4.8)
Bilirubin, mg/dL	
≤1.9	76 351 (94.3)
2.0-2.9	2603 (3.2)
3.0-4.9	1197 (1.5)
5.0-7.9	469 (0.6)
≥ 8.0	340 (0.4)
Blood urea nitrogen, mg/dL	
<16.9	12 071 (14.9)
17-19	6558 (8.1)
20-39	37 620 (46.5)
40-79	19 713 (24.4)
≥80	4998 (6.2)
Hematocrit, %	
41-49	73 736 (91.1)
<40.9 or ≥50	7224 (8.9)

Abbreviations: ACEI, angiotensin converting enzyme inhibitor; ARB, angiotensin receptor blocker; SBP, systolic blood pressure.

SI conversion factors: To convert albumin to g/L, multiply by 10; bilirubin to micromoles per liter, multiply by 17.104; serum creatinine to micromoles per liter, multiply by 88.4; and blood urea nitrogen to millimoles per liter, multiply by 0.357.

^a^ Defined as a creatinine within 10% of the baseline value.

### Mortality

Adjusted HRs (aHRs) for each blood pressure category and time point relative to SBP of 160 mm Hg or greater are given in Table 2. Patients with SBP between 130 and 139 mm Hg had the lowest risk for mortality at most time points, and the mediation of timing was most evident in this group, which had a statistically significant increase in mortality risk relative to patients with SBP of 160 mm Hg or greater at 60 days (aHR, 1.20; 99% CI, 1.00-1.44); no association with mortality at 90 days, 120 days, and 180 days; and a statistically significant decrease in mortality risk by 270 days (aHR, 0.72; 99% CI, 0.59-0.90) that persisted at 365 days (aHR, 0.58; 99% CI, 0.45-0.76). Patients with SBP less than 120 mm Hg had the highest risk of mortality at each time point including at 60 days (aHR, 2.20; 99% CI, 1.85-2.62) and 365 days (aHR, 1.82; 99% CI, 1.47-2.25). Notably, patients with SBP between 120 and 129 mm Hg had significantly higher risk of mortality relative to patients with SBP of 160 mm Hg or greater through the first 180 days, with no statistical differences between these groups seen at 270 or 365 days.

**Table 2.** Mortality and All-Cause Hospital Readmission Over Time by Blood Pressure Category^a^

Days from hospital discharge	Patients by systolic blood pressure, mm Hg										
	<120, AHR (99% CI)	*P* value	120-129, AHR (99% CI)	*P* value	130-139, AHR (99% CI)	*P* value	140-149, AHR (99% CI)	*P* value	150-159, AHR (99% CI)	*P* value	≥160
Mortality											
60	2.20 (1.85-2.62)	<.001	1.36 (1.14-1.63)	<.001	1.20 (1.00-1.44)	.01	1.11 (0.90-1.36)	.20	1.25 (0.98-1.58)	.02	1 [Reference]
90	2.16 (1.82-2.56)	<.001	1.31 (1.10-1.57)	<.001	1.12 (0.93-1.33)	.12	1.08 (0.89-1.32)	.31	1.18 (0.94-1.47)	.06	1 [Reference]
120	2.12 (1.79-2.50)	<.001	1.27 (1.07-1.51)	<.001	1.04 (0.87-1.24)	.58	1.06 (0.87-1.28)	.48	1.11 (0.89-1.39)	.22	1 [Reference]
180	2.04 (1.72-2.42)	<.001	1.19 (0.99-1.42)	.01	0.90 (0.75-1.08)	.13	1.01 (0.82-1.23)	.94	0.99 (0.78-1.26)	.99	1 [Reference]
270	1.93 (1.60-2.32)	.001	1.07 (0.87-1.31)	.39	0.72 (0.59-0.90)	<.001	0.94 (0.73-1.20)	.49	0.83 (0.61-1.14)	.14	1 [Reference]
365	1.82 (1.47-2.25)	<.001	0.97 (0.76-1.23)	.71	0.58 (0.45-0.76)	<.001	0.87 (0.64-1.19)	.26	0.70 (0.46-1.06)	.03	1 [Reference]
Readmission											
60	1.28 (1.18-1.40)	<.001	1.15 (1.05-1.25)	<.001	1.07 (0.98-1.18)	.04	1.13 (1.02-1.25)	.001	1.14 (1.02-1.28)	.003	Reference
90	1.21 (1.11-1.32)	<.001	1.09 (1.00-1.19)	.01	1.02 (0.93-1.11)	.63	1.08 (0.98-1.19)	.04	1.10 (0.99-1.23)	.03	1 [Reference]
120	1.14 (1.04-1.24)	<.001	1.03 (0.95-1.13)	.31	0.96 (0.88-1.05)	.27	1.03 (0.94-1.13)	.42	1.06 (0.95-1.18)	.17	1 [Reference]
180	1.01 (0.92-1.10)	.80	0.93 (0.85-1.03)	.06	0.86 (0.79-0.95)	<.001	0.94 (0.85-1.04)	.12	0.98 (0.87-1.11)	.74	1 [Reference]
270	0.84 (0.76-0.94)	.001	0.80 (0.72-0.90)	<.001	0.73 (0.66-0.82)	<.001	0.82 (0.72-0.93)	<.001	0.88 (0.75-1.04)	.05	1 [Reference]
365	0.70 (0.61-0.80)	<.001	0.69 (0.60-0.79)	<.001	0.62 (0.54-0.72)	<.001	0.71 (0.60-0.85)	<.001	0.79 (0.63-0.99)	.006	1 [Reference]

Abbreviation: AHR, adjusted hazard ratio.

^a^ Models were adjusted for age, comorbidities (chronic lung disease, congestive heart failure, dementia, malignant neoplasm, and unexplained weight loss), laboratory values (hematocrit, blood urea nitrogen, bilirubin, and albumin), and kidney-specific variables (acute kidney injury stage, community-acquired vs in-hospital acute kidney injury, proteinuria, baseline creatinine, and discharge creatinine).

**Figure 2.**
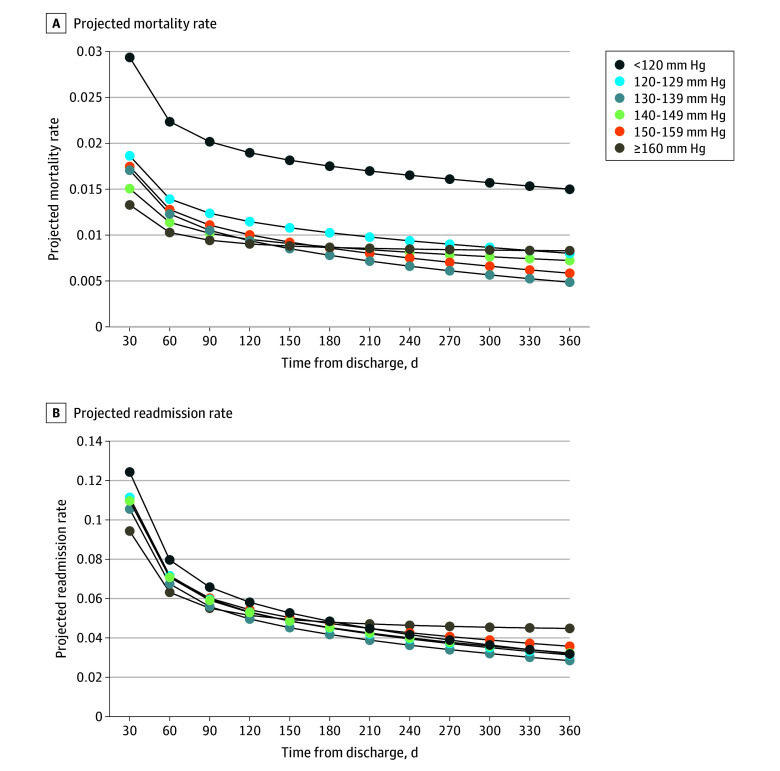
Projected Monthly Mortality Rates and All-Cause Readmission Rates Over Time From Hospital Discharge The figure shows projected monthly mortality rates (A) and all-cause readmission rates (B) over time from hospital discharge for all blood pressure categories for an average patient with all variables in the model except the blood pressure category set to mean values.

Projected mortality rates within each SBP group for the average patient are shown in Figure 2A. Patients with SBP less than 120 mm Hg had the highest projected mortality at each time point. Patients with SBP of 160 mm Hg or greater had the lowest projected mortality at 60 days but had higher projected mortality than all groups except those with SBP less than 120 mm Hg at 365 days. Monthly absolute mortality rates by most recent SBP category showed a similar pattern (eFigure 1 in Supplement 1).

### All-Cause Readmission

Calculated aHRs for each blood pressure category and time point are given in Table 2. Patients with SBP between 130 and 139 mm Hg had the lowest risk of readmission at most time points, with significantly lower risk of readmission by 180 days. Patients with SBP between 130 and 139 mm Hg were also the only group that did not have a statistically higher risk for readmission at 60 days relative to patients with SBP of 160 mm Hg or greater. Figure 2B shows the projected rates of hospital readmission at each time point and shows that patients with SBP of 160 mm Hg or greater had the lowest projected readmission rate initially, but the highest rate at every time point after approximately the 6-month mark. Monthly absolute readmission rates are shown in eFigure 2 in Supplement 1.

### ACEI and ARB Analysis

The overall risks for both mortality and readmission were similar in the analysis stratified by ACEI and ARB use at 60 days, with lower blood pressures associated with higher risk of mortality and readmission (eTable 2 in Supplement 1). Patients with SBP less than 120 mm Hg, between 120 and 129 mm Hg, between 130 and 139 mm Hg, and 160 mm Hg or greater had significantly lower relative risks of mortality with ACEI or ARB use compared with those without ACEI or ARB use at 60 days (Table 3). These benefits lasted to the 180-day mark for patients with SBP between 130 and 139 mm Hg. There were no statistically significant differences in risks of readmission in any blood pressure group with ACEI or ARB use compared with no ACEI or ARB use. Graphs of projected risks of mortality by SBP category over time by ACEI and ARB status are shown in eFigure 3 in Supplement 1.

**Table 3.** Mortality and All-Cause Readmission Over Time for Patients Taking vs Not Taking Angiotensin Converting Enzyme Inhibitor or Angiotensin Receptor Blocker at 60 Days From Discharge^a^

Days from hospital discharge	Patients by systolic blood pressure, mm Hg											
	<120, AHR (99% CI)	*P* value	120-129, AHR (99% CI)	*P* value	130-139, AHR (99% CI)	*P* value	140-149, AHR (99% CI)	*P* value	150-159, AHR (99% CI)	*P* value	≥160, AHR (99%CI)	*P* value
Mortality												
60	0.84 (0.75-0.94)	.001	0.86 (0.73-1.00)	.01	0.83 (0.69-0.98)	.005	0.89 (0.69-1.14)	.23	0.82 (0.58-1.16)	.14	0.73 (0.52-1.03)	.02
180	0.95 (0.85-1.06)	.23	0.89 (0.76-1.04)	.047	0.84 (0.70-1.00)	.009	1.01 (0.80-1.28)	.90	0.86 (0.62-1.19)	.22	0.78 (0.55-1.10)	.06
365	1.06 (0.83-1.35)	.54	0.86 (0.61-1.23)	.28	0.79 (0.53-1.18)	.13	1.13 (0.67-1.91)	.54	0.84 (0.40-1.78)	.56	0.79 (0.56-1.12)	.08
Readmission												
60	1.01 (0.94-1.09)	.63	0.93 (0.85-1.01)	.02	0.95 (0.87-1.04)	.13	1.02 (0.91-1.15)	.63	0.96 (0.81-1.13)	.48	0.88 (0.73-1.05)	.06
180	0.94 (0.86-1.02)	.04	0.92 (0.83-1.01)	.02	0.96 (0.87-1.05)	.24	1.02 (0.90-1.17)	.64	0.89 (0.74-1.07)	.11	0.87 (0.72-1.04)	.04
365	0.84 (0.69-1.02)	.02	0.91 (0.73-1.14)	.28	0.98 (0.79-1.22)	.81	1.04 (0.77-1.41)	.74	0.82 (0.54-1.24)	.21	0.87 (0.72-1.04)	.04

Abbreviation: AHR, adjusted hazard ratio.

^a^ Models were adjusted for age, comorbidities (chronic lung disease, congestive heart failure, dementia, malignancy, and unexplained weight loss), laboratory values (hematocrit, blood urea nitrogen, bilirubin, and albumin), and kidney-specific variables (acute kidney injury stage, community-acquired vs in-hospital acute kidney injury, proteinuria, baseline creatinine, and discharge creatinine).

### CHF Sensitivity Analysis

HRs for mortality and all-cause hospital readmission for those without a prior history of CHF are given in eTable 3 in Supplement 1. Similar associations of SBP with both mortality and readmission over time were observed in the non-CHF cohort. The most notable difference was in the 130 to 139 mm Hg group, which did not show increased risk of mortality at 60 days as in the overall cohort.

### AKI Severity Sensitivity Analysis

HRs for mortality and all-cause hospital readmission in those with stage 1 AKI and in those with stage 2 or 3 AKI, including those receiving renal replacement therapy are given in eTable 4 in Supplement 1. There were no major differences observed in the association of blood pressure with mortality or blood pressure with readmission over time based on AKI severity, which was consistent with our previously published findings.^5^

## Discussion

In this cohort study of patients post-AKI, we found that associations of SBP with both mortality and hospital readmission rates were substantially affected by timing relative to hospital discharge. Risks of mortality and hospital readmission in all SBP groups relative to the 160 mm Hg or greater group were highest immediately following discharge and decreased over time. Patients with SBP between 130 and 139 mm Hg had the lowest overall risk for mortality and readmission among the groups evaluated. Patients with SBP less than 120 mm Hg had the worst overall risk profile in this population, including the highest risk for mortality at each time point. Our sensitivity analyses showed similar associations of SBP with mortality and readmission, regardless of CHF status or AKI stage. Finally, similar risks for mortality and readmission were observed in those with and without use of an ACEI or ARB medication, with lower relative risks in those with ACEI or ARB use within 60 days. These findings may have substantial implications for patient care following an in-hospital AKI event.

Recent studies^2,28^ have demonstrated a clear association of AKI with long-term mortality, especially CVD-related mortality. As a result, many post-AKI clinics have made blood pressure control a focus^27^ based on both the high rates of CVD risk factors in the post-AKI population and from evidence, such as the SPRINT trial,^8^ showing benefit from intensive blood pressure control in populations with CKD. However, data to support specific blood pressure goals in the post-AKI population are sparse, and existing studies^10,11,12^ suggest the possibility of higher short-term complications related to intensive blood pressure lowering. Even within SPRINT,^8^ rates of AKI were higher in the intensive control group, a complication which may be more pronounced and more detrimental in the post-AKI population. Our study substantially adds to the literature by looking at over 80 000 patients post-AKI, a large population that allowed us to look at a greater number of SBP groups, to evaluate risks at multiple time points, and to use multiple blood pressure readings over time. While caution must be exercised when applying observational studies to clinical practice, our results would support a strategy of allowing for a 3-month recovery period prior to escalating blood pressure regimens to target a blood pressure of 130 to 139 mm Hg in those who experienced AKI during a hospital admission.

Our findings also provide additional nuance to questions regarding ACEI and ARB use in the postdischarge period. We did observe some lower relative risks of mortality in those with ACEI or ARB use at 60 days, as shown in Table 3. It should be noted, however, that in the case of patients with SBP less than 120 mm Hg or between 120 and 129 mm Hg, there were lower relative risks of mortality with ACEI and ARB use, but overall higher mortality rates at 60 and 180 days compared with patients with higher blood pressures (Table 2 and eTable 2 in Supplement 1). In addition, the results did not show increased risk for readmission in those receiving ACEI or ARB relative to those not receiving ACEI or ARB, which is consistent with the findings of a previous large observational study.^16^ Our study, therefore, adds to the existing literature by showing that ACEI and ARB risks and benefits potentially do not apply equally at all blood pressures and time points, and also by raising the possibility that overall blood pressure ranges may be more clinically significant in the post-AKI population than ACEI and ARB use, although further prospective research is needed.

### Limitations

This study has several limitations. Due to its retrospective design, residual confounding could not be excluded despite our attempts to appropriately adjust the model. We also did not have adequate data available to determine whether medication regiments were adjusted in this population to achieve blood pressure goals. The use of retrospective data meant that some patients were seen more frequently in follow-up than others, which could have generated ascertainment bias and introduced bias related to level of medical care. The transition from the *International Classification of Diseases, Ninth Revision (ICD-9)* to *International Statistical Classification of Diseases and Related Health Problems, Tenth Revision (ICD-10)* may have impacted ascertainment of comorbid conditions including CHF. Finally, the use of VHA data resulted in a population that is overwhelmingly male, limiting generalizability to female patients. Notwithstanding these limitations, the study has several strengths. The model was developed in a large, racially diverse cohort of adults hospitalized with AKI. Use of VHA data also increased the probability that a complete record of post-AKI blood pressure measurements was available for all patients and allowed us to model blood pressure as a time-dependent covariate, an approach that is likely superior to use of blood pressure at a single time point.

## Conclusions

In this retrospective cohort study of patients post-AKI, there were substantial variations in the associations of SBP with mortality based on time from discharge. Risks of mortality and readmission relative to elevated blood pressure were highest in the immediate postdischarge period, with a shift toward lower mortality and readmission at later time points. Veterans with SBP between 130 and 139 mm Hg had the most favorable risk level over time of any group. These findings may have important implications for the timing and targets for blood pressure control used in post-AKI care.

## Notes

Supplement 1. **eTable 1.** Patient Characteristics Based on First Blood Pressure After Discharge **eFigure 1.** Unadjusted Absolute Mortality Rates Over Time From Hospital Discharge for All Blood Pressure Categories **eFigure 2.** Unadjusted Absolute Readmission Rates Over Time From Hospital Discharge for All Blood Pressure Categories **eTable 2.** ACEI/ARB Stratification: Hazard Ratios and 99% Confidence Intervals for Mortality and Readmission Over Time, by Blood Pressure Category **eFigure 3.** Predicted Monthly Mortality Rates Over Time From Hospital Discharge for an “Average” Patient, With All Variables in the Model Except the Blood Pressure Category Set to Mean Values, for All Blood Pressure Categories in Those Who Did and Did Not Have an ACEI/ARB Prescribed Within 90 Days **eTable 3.** In Patients Without CHF at Baseline, Adjusted Hazard Ratios and 99% Confidence Intervals for Mortality and All-Cause Hospital Readmission Over Time, by Blood Pressure Category **eTable 4.** AKI Stage Stratification: Hazard Ratios and 95% Confidence Intervals for Mortality and Readmission Over Time, by Blood Pressure Category

Supplement 2.
Data Sharing Statement
