# Supplementary material for: Blood Pressure, Readmission, and Mortality Among Patients Hospitalized With Acute Kidney Injury
Source: JAMA Netw Open. 2024 May 13;7(5):e2410824. doi: 10.1001/jamanetworkopen.2024.10824 (PMC11091759; doi:10.1001/jamanetworkopen.2024.10824)
Supplement: Supplement 1. — eTable 1. Patient Characteristics Based on First Blood Pressure After Discharge eFigure 1. Unadjusted Absolute Mortality Rates Over Time From Hospital Discharge for All Blood Pressure Categories eFigure 2. Unadjusted Absolute Readmission Rates Over Time From Hospital Discharge for All Blood Pressure Categories eTable 2. ACEI/ARB Stratification: Hazard Ratios and 99% Confidence Intervals for Mortality and Readmission Over Time, by Blood Pressure Category eFigure 3. Predicted Monthly Mortality Rates Over Time From Hospital Discharge for an “Average” Patient, With All Variables in the Model Except the Blood Pressure Category Set to Mean Values, for All Blood Pressure Categories in Those Who Did and Did Not Have an ACEI/ARB Prescribed Within 90 Days eTable 3. In Patients Without CHF at Baseline, Adjusted Hazard Ratios and 99% Confidence Intervals for Mortality and All-Cause Hospital Readmission Over Time, by Blood Pressure Category eTable 4. AKI Stage Stratification: Hazard Ratios and 95% Confidence Intervals for Mortality and Readmission Over Time, by Blood Pressure Category [file jamanetwopen-e2410824-s001.pdf]

Supplemental Online Content

Griffin BR, Vaughan-Sarrazin M, Shi Q, et al. Blood pressure, readmission, and mortality among patients hospitalized with acute kidney injury. *JAMA Netw Open*. 2024;7(5):e2410824. doi:10.1001/jamanetworkopen.2024.10824

eTable 1. Patient Characteristics Based on First Blood Pressure After Discharge

eFigure 1. Unadjusted Absolute Mortality Rates Over Time From Hospital Discharge for All Blood Pressure Categories

eFigure 2. Unadjusted Absolute Readmission Rates Over Time From Hospital Discharge for All Blood Pressure Categories

eTable 2. ACEI/ARB Stratification: Hazard Ratios and 99% Confidence Intervals for Mortality and Readmission Over Time, by Blood Pressure Category

eFigure 3. Predicted Monthly Mortality Rates Over Time From Hospital Discharge for an “Average” Patient, With All Variables in the Model Except the Blood Pressure Category Set to Mean Values, for All Blood Pressure Categories in Those Who Did and Did Not Have an ACEI/ARB Prescribed Within 90 Days

eTable 3. In Patients Without CHF at Baseline, Adjusted Hazard Ratios and 99% Confidence Intervals for Mortality and All-Cause Hospital Readmission Over Time, by Blood Pressure Category

eTable 4. AKI Stage Stratification: Hazard Ratios and 95% Confidence Intervals for Mortality and Readmission Over Time, by Blood Pressure Category

This supplemental material has been provided by the authors to give readers additional information about their work.

© 2024 Griffin BR et al. *JAMA Network Open.*

eTable 1. Patient Characteristics Based on First Blood Pressure After Discharge

Characteristics*		<120<br>n=24,074	120-129<br>N=20,063	130-139<br>N=18,678	140-149<br>N=9,995	150-159<br>N=4,887	≥160<br>N=3,263	p-value
Age (years)								<.001
	<45	546 (2.3)	472 (2.4)	410 (2.2)	143 (1.4)	65 (1.3)	26 (0.8)	
	45-59	3,050 (12.7)	2,844 (14.2)	2,560 (13.7)	1,394 (13.9)	634 (13.0)	428 (13.1)	
	60-64	3,247 (13.5)	2,814 (14.0)	2,546 (13.6)	1,394 (13.9)	697 (14.3)	448 (13.7)	
	65-69	5,618 (23.3)	4,562 (22.7)	4,415 (23.6)	2,325 (23.3)	1,174 (24.0)	779 (23.9)	
	70-74	4,385 (18.2)	3,504 (17.5)	3,351 (17.9)	1,818 (18.2)	895 (18.3)	570 (17.5)	
	75-84	4,597 (19.1)	3,800 (18.9)	3,531 (18.9)	1,829 (18.3)	925 (18.9)	641 (19.6)	
	>84	2,631 (10.9)	2,067 (10.3)	1,865 (10.0)	1,092 (10.9)	497 (10.2)	371 (11.4)	
Black Race		4,490 (18.9)	4,595 (19.1)	4,418 (22.0)	4,398 (23.5)	2,619 (26.2)	1,377 (28.2)	<.001
ACEI/ARB use within 60 days of discharge		10,041 (41.7)	8,366 (41.7)	8,090 (43.3)	4,713 (47.2)	2,370 (48.5)	1,719 (52.7)	<.001
Unexplained Weight Loss		1,941 (8.1)	1,274 (6.3)	1,061 (5.7)	552 (5.5)	254 (5.2)	174 (5.3)	<.001
Dementia		1,285 (5.3)	1,026 (5.1)	918 (4.9)	483 (4.8)	249 (5.1)	154 (4.7)	0.3
Congestive Heart Failure		8,383 (34.8)	5,314 (26.5)	4,412 (23.6)	2,491 (24.9)	1,208 (24.7)	808 (24.8)	<.001
Chronic Lung Disease		9,184 (38.1)	6,893 (34.4)	6,082 (32.6)	3,094 (31.0)	1,509 (30.9)	920 (28.2)	<.001
Baseline serum creatinine, mean (SD)		1.3 ± 0.6	1.3 ± 0.7	1.3 ± 0.8	1.4 ± 0.9	1.5 ± 1.0	1.7 ± 1.1	<.001
Discharge serum creatinine, mean (SD)		1.4 ± 1.2	1.5 ± 1.3	1.6 ± 1.4	1.7 ± 1.6	1.8 ± 1.6	2.0 ± 1.8	<.001
Renal Replacement Therapy During Admission		231 (1.0)	211 (1.1)	208 (1.1)	152 (1.5)	79 (1.6)	57 (1.8)	<.001
Tobacco Use		5439 (22.5)	4345 (21.7)	3858 (20.7)	2060 (20.6)	984 (20.1)	625 (19.2)	<.001
Albumin (g/g)								<.001
	≤ 1.9	590 (2.5)	405 (2.0)	348 (1.9)	211 (2.1)	91 (1.9)	84 (2.6)	
	2.0 -2.4	1,767 (7.3)	1,290 (6.4)	1,148 (6.1)	624 (6.2)	341 (7.0)	239 (7.3)	
	2.5 – 4.4	20,676 (85.9)	17,314 (86.3)	16,227 (86.9)	8,699 (87.0)	4,234 (86.6)	2,812 (86.2)	
	≥ 4.5	1,041 (4.3)	1,054 (5.3)	955 (5.1)	461 (4.6)	221 (4.5)	128 (3.9)	
Bilirubin (mg/dL)								<.001
	≤ 1.9	22,312 (92.7)	18,875 (94.1)	17,729 (94.9)	9,560 (95.6)	4,713 (96.4)	3,162 (96.9)	
	2.0 - 2.9	959 (4.0)	699 (3.5)	542 (2.9)	233 (2.3)	107 (2.2)	63 (1.9)	
	3.0 – 4.9	464 (1.9)	306 (1.5)	249 (1.3)	111 (1.1)	41 (0.8)	26 (0.8)	
	5.0 – 7.9	202 (0.8)	89 (0.4)	101 (0.5)	53 (0.5)	17 (0.3)	7 (0.2)	
	≥ 8.0	137 (0.6)	94 (0.5)	57 (0.3)	38 (0.4)	9 (0.2)	5 (0.2)	
Blood Urea Nitrogen (mg/dL)								<.001
	< 16.9	3,699 (15.4)	3,262 (16.3)	2,826 (15.1)	1,375 (13.8)	566 (11.6)	343 (10.5)	
	17-19	1,939 (8.1)	1,699 (8.5)	1,558 (8.3)	780 (7.8)	369 (7.6)	213 (6.5)	
	20-39	11,248 (46.7)	9,411 (46.9)	8,711 (46.6)	4,527 (45.3)	2,247 (46.0)	1,476 (45.2)	
	40-79	5,697 (23.7)	4,563 (22.7)	4,492 (24.0)	2,639 (26.4)	1,348 (27.6)	974 (29.8)	
	≥ 80	1,491 (6.2)	1,128 (5.6)	1,091 (5.8)	674 (6.7)	357 (7.3)	257 (7.9)	
Hematocrit								<.001
	41-49%	21,751 (90.4)	18,374 (91.6)	16,982 (90.9)	9,138 (91.4)	4,465 (91.4)	3,026 (92.7)	
	<40.9% OR ≥50%	2,323 (9.6)	1,689 (8.4)	1,696 (9.1)	857 (8.6)	422 (8.6)	237 (7.3)	
AKI Stage								<.001
	1	19,301 (80.2)	15,843 (79.0)	14,672 (78.6)	7,659 (76.6)	3,700 (75.7)	2,413 (74.0)	
	2	2,448 (10.2)	2,053 (10.2)	1,836 (9.8)	941 (9.4)	432 (8.8)	245 (7.5)	
	3	2,325 (9.7)	2,167 (10.8)	2,170 (11.6)	1,395 (14.0)	755 (15.4)	605 (18.5)	
Proteinuria during hospital episode								<.001
	normal	8187 (34.0)	6332 (31.6)	5255 (28.1)	2528 (25.3)	1118 (22.9)	677 (20.8)	
	mild	11168 (46.4)	9597 (47.8)	8990 (48.1)	4848 (48.5)	2285 (46.8)	1560 (47.8)	
	heavy	2492 (10.4)	2545 (12.7)	2894 (15.5)	1847 (18.5)	1123 (23.0)	758 (23.2)	
	missing	2227 (9.3)	1589 (7.9)	1539 (8.2)	772 (7.7)	361 (7.4)	268 (8.2)	

© 2024 Griffin BR et al. *JAMA Network Open.*

eFigure 1. Unadjusted Absolute Mortality Rates Over Time From Hospital Discharge for All Blood Pressure Categories

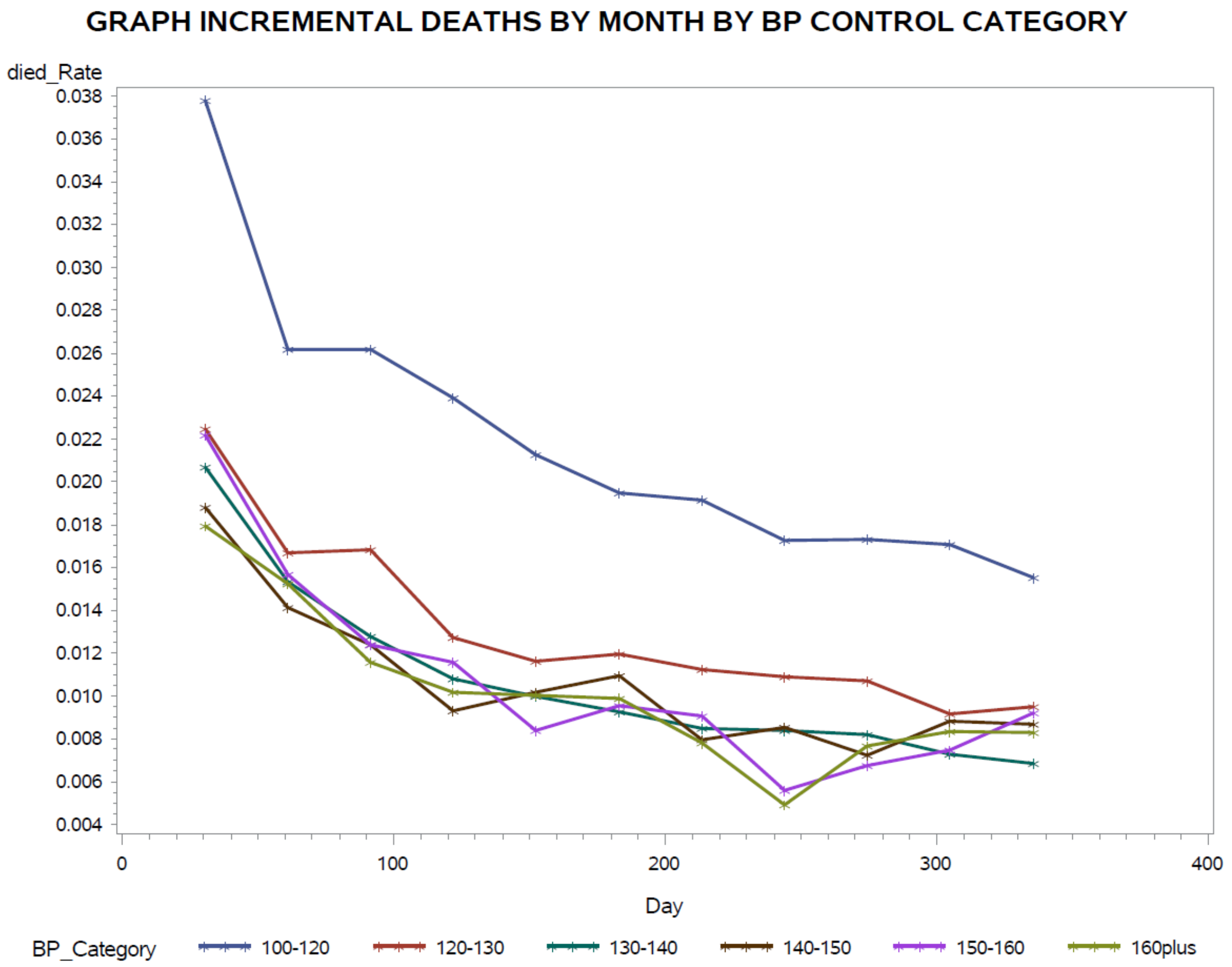

© 2024 Griffin BR et al. *JAMA Network Open.*

eFigure 2. Unadjusted Absolute Readmission Rates Over Time From Hospital Discharge for All Blood Pressure Categories

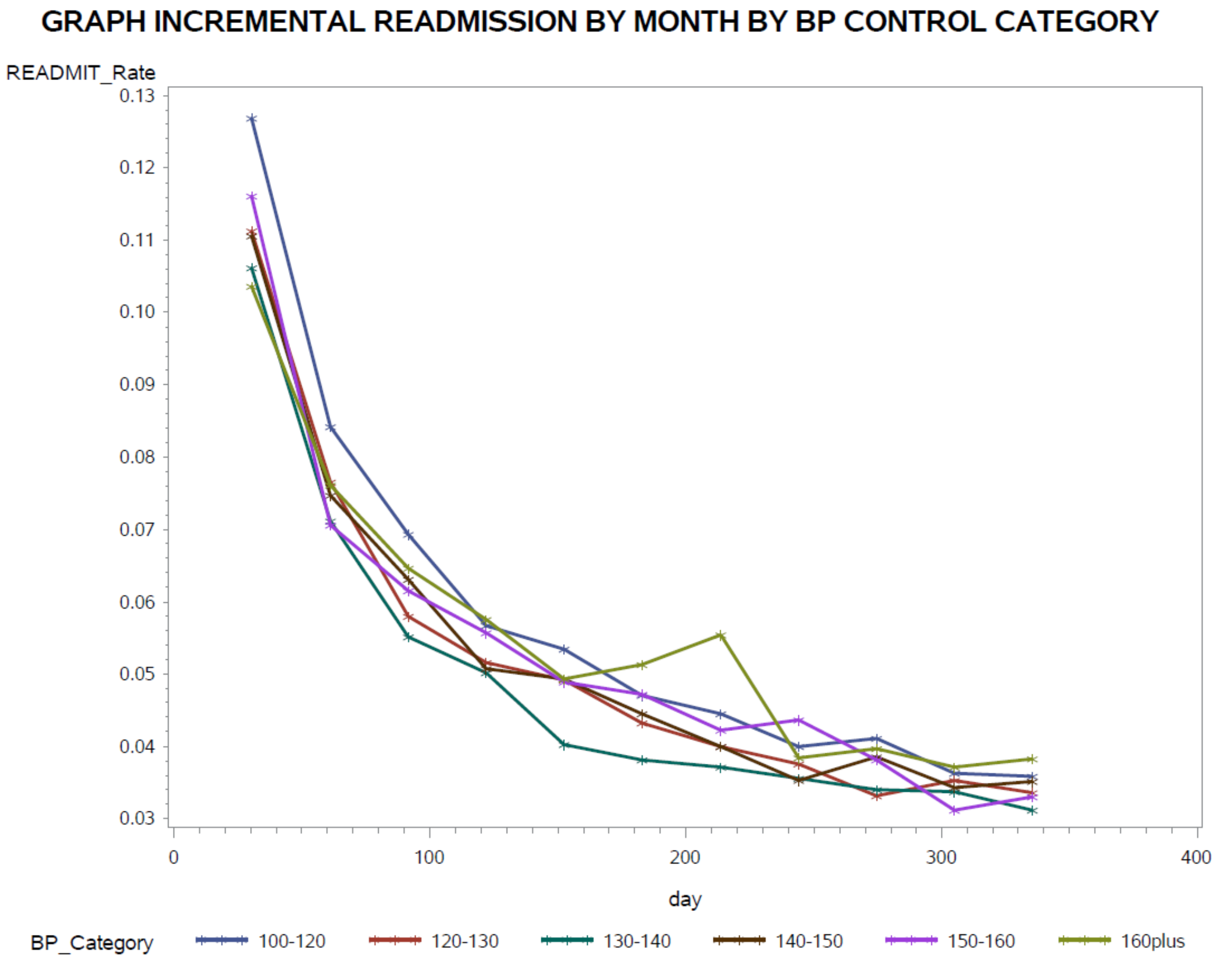

© 2024 Griffin BR et al. *JAMA Network Open.*

eTable 2. ACEI/ARB Stratification: Hazard Ratios and 99% Confidence Intervals for Mortality and Readmission Over Time, by Blood Pressure Category

A. No ACEI/ARB Within 60 Days

			Systolic Blood Pressure (mm Hg)					
			**<120**	**120-129**	**130-139**	**140-149**	**150-159**	**≥160**
**Days From Hospital Discharge**	**Mortality**	**60**	1.97 (1.45-2.66)<br>p < 0.001	1.20 (0.88-1.64)<br>p = 0.1	1.08 (0.78-1.48)<br>p = 0.5	0.95 (0.67-1.36)<br>p = 0.7	1.13 (0.76-1.69)<br>p = 0.4	Reference
		**90**	1.91 (1.42-2.58)<br>p < 0.001	1.17 (0.86-1.58)<br>p = 0.2	1.01 (0.74-1.38)<br>p = 0.9	0.92 (0.65-1.30)<br>p = 0.5	1.07 (0.73-1.58)<br>p = 0.6	
		**120**	1.86 (1.39-2.50)<br>p < 0.001	1.13 (0.83-1.53)<br>p = 0.3	0.94 (0.69-1.29)<br>p = 0.6	0.89 (0.63-1.25)<br>p = 0.4	1.02 (0.69-1.49)<br>p = 0.9	
		**180**	1.77 (1.31-2.38)<br>p < 0.001	1.06 (0.78-1.45)<br>p = 0.6	0.83 (0.60-1.14)<br>p = 0.1	0.83 (0.58-1.18)<br>p = 0.2	0.91 (0.60-1.39)<br>p = 0.6	
		**270**	1.63 (1.19-2.24)<br>p < 0.001	0.97 (0.69-1.37)<br>p = 0.8	0.68 (0.47-0.98)<br>p = 0.006	0.74 (0.49-1.14)<br>p = 0.07	0.78 (0.45-1.34)<br>p = 0.2	
		**365**	1.51 (1.05-2.15)<br>p = 0.003	0.89 (0.59-1.33)<br>p = 0.4	0.56 (0.36-0.86)<br>p < 0.001	0.67 (0.39-1.14)<br>p = 0.051	0.66 (0.32-1.36)<br>p = 0.1	
	**Readmission**	**60**	1.18 (1.00-1.39)<br>p = 0.011	1.20 (0.88-1.64)<br>p = 0.1	1.21 (0.96-1.52)<br>p = 0.03	1.16 (0.90-1.49)<br>p = 0.1	1.27 (0.95-1.70)<br>p=0.03	Reference
		**90**	1.12 (0.95-1.32)<br>p = 0.07	1.17 (0.86-1.58)<br>p = 0.2	1.12 (0.90-1.40)<br>p = 0.2	1.14 (0.89-1.45)<br>p = 0.2	1.20 (0.91-1.58)<br>p = 0.09	
		**120**	1.07 (0.91-1.26)<br>p = 0.3	1.13 (0.83-1.53)<br>p = 0.3	1.04 (0.84-1.29)<br>p = 0.6	1.12 (0.88-1.42)<br>p = 0.2	1.13 (0.86-1.48)<br>p = 0.3	
		**180**	0.97 (0.82-1.14)<br>p = 0.6	1.06 (0.78-1.45)<br>p = 0.6	0.89 (0.71-1.12)<br>p = 0.2	1.08 (0.84-1.38)<br>p = 0.4	1.00 (0.75-1.34)<br>p > 0.99	
		**270**	0.83 (0.69-1.01)<br>p = 0.014	0.97 (0.69-1.37)<br>p = 0.8	0.71 (0.54-0.93)<br>p = 0.001	1.02 (0.75-1.39)<br>p = 0.9	0.84 (0.57-1.23)<br>p = 0.2	
		**365**	0.72 (0.57-0.90)<br>p < 0.001	0.89 (0.59-1.33)<br>p = 0.4	0.57 (0.41-0.79)<br>p < 0.001	0.97 (0.65-1.43)<br>p = 0.8	0.70 (0.42-1.16)<br>p = 0.07	

B. ACEI/ARB Within 60 Days

			Systolic Blood Pressure (mm Hg)					
			**<120**	**120-129**	**130-139**	**140-149**	**150-159**	**≥160**
**Days From Hospital Discharge**	**Mortality**	**60**	2.19 (1.77-2.70)<br>p < 0.001	1.37 (1.10-1.71)<br>p < 0.001	1.02 (0.86-1.21)<br>p = 0.8	1.02 (0.85-1.23)<br>p = 0.8	1.07 (0.87-1.33)<br>p = 0.4	Reference
		**90**	2.16 (1.76-2.66)<br>p < 0.001	1.32 (1.06-1.64)<br>p = 0.001	0.96 (0.82-1.14)<br>p = 0.6	0.97 (0.81-1.16)<br>p = 0.7	1.04 (0.85-1.28)<br>p = 0.6	
		**120**	2.13 (1.74-2.61)<br>p < 0.001	1.27 (1.03-1.57)<br>p = 0.003	0.91 (0.77-1.08)<br>p = 0.2	0.92 (0.77-1.11)<br>p = 0.3	1.02 (0.83-1.25)<br>p = 0.8	
		**180**	2.08 (1.69-2.55)<br>p < 0.001	1.18 (0.95-1.47)<br>p = 0.048	0.81 (0.68-0.97)<br>p = 0.002	0.84 (0.69-1.02)<br>p = 0.019	0.96 (0.77-1.21)<br>p = 0.7	
		**270**	2.00 (1.59-2.51)<br>p < 0.001	1.06 (0.82-1.36)<br>p = 0.6	0.69 (0.56-0.84)<br>p < 0.001	0.72 (0.57-0.92)<br>p = 0.001	0.89 (0.66-1.21)<br>p = 0.3	
		**365**	1.92 (1.47-2.51)<br>p < 0.001	0.95 (0.69-1.29)<br>p = 0.6	0.58 (0.45-0.74)<br>p < 0.001	0.63 (0.46-0.85)<br>p < 0.001	0.82 (0.55-1.23)<br>p = 0.2	
	**Readmission**	**60**	1.32 (1.19-1.46)<br>p < 0.001	1.14 (1.02-1.27)<br>p = 0.002	1.08 (0.97-1.20)<br>p = 0.06	1.17 (1.04-1.32)<br>p < 0.001	1.16 (1.01-1.32)<br>p = 0.005	Reference
		**90**	1.23 (1.11-1.36)<br>p < 0.001	1.08 (0.98-1.20)<br>p = 0.051	1.02 (0.92-1.14)<br>p = 0.5	1.12 (1.00-1.26)<br>p = 0.008	1.11 (0.98-1.27)<br>p = 0.03	
		**120**	1.15 (1.04-1.27)<br>p < 0.001	1.03 (0.93-1.14)<br>p = 0.5	0.97 (0.88-1.08)<br>p = 0.5	1.07 (0.96-1.20)<br>p = 0.1	1.07 (0.94-1.22)<br>p = 0.2	
		**180**	1.00 (0.90-1.11)<br>p > 0.99	0.93 (0.83-1.04)<br>p = 0.09	0.87 (0.78-0.98)<br>p = 0.002	0.98 (0.87-1.11)<br>p = 0.7	0.98 (0.85-1.14)<br>p=0.8	
		**270**	0.81 (0.71-0.93)<br>p < 0.001	0.80 (0.69-0.92)<br>p < 0.001	0.75 (0.65-0.86)<br>p < 0.001	0.86 (0.73-1.01)<br>p = 0.02	0.87 (0.71-1.06)<br>p = 0.08	
		**365**	0.66 (0.56-0.79)<br>p < 0.001	0.68 (0.57-0.82)<br>p < 0.001	0.64 (0.54-0.76)<br>p < 0.001	0.76 (0.61-0.93)<br>p = 0.001	0.77 (0.59-1.01)<br>p = 0.013	

Models were adjusted for age, comorbidities (chronic lung disease, congestive heart failure, dementia, malignancy, unexplained weight loss), laboratory values (hematocrit, blood urea nitrogen, bilirubin, albumin), and kidney-specific variables (AKI stage, community-acquired vs in-hospital AKI, proteinuria during hospital episode, baseline creatinine, discharge creatinine).

© 2024 Griffin BR et al. *JAMA Network Open.*

eFigure 3. Predicted Monthly Mortality Rates Over Time From Hospital Discharge for an "Average" Patient, With All Variables in the Model Except the Blood Pressure Category Set to Mean Values, for All Blood Pressure Categories in Those Who Did and Did Not Have an ACEI/ARB Prescribed Within 90 Days

A. No ACEI/ARB Use

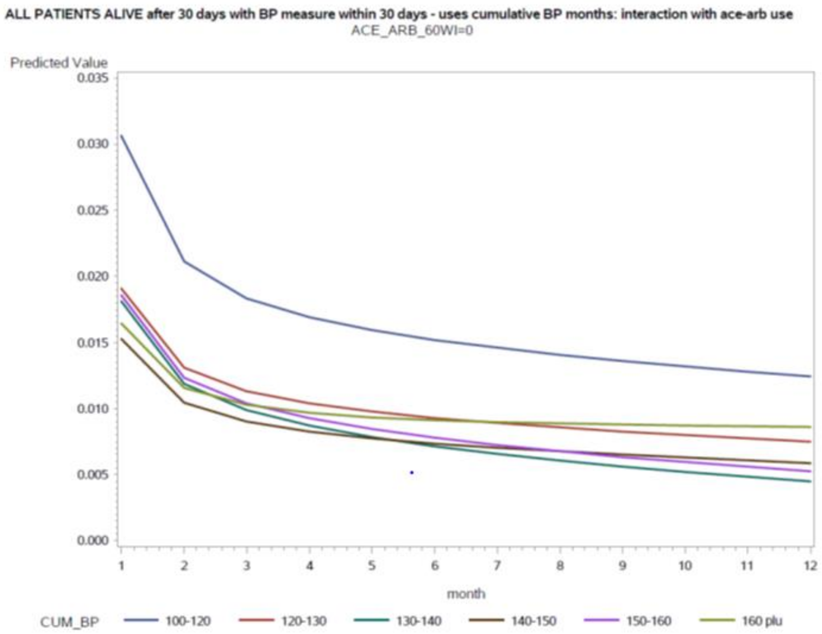

B. ACEI/ARB Use

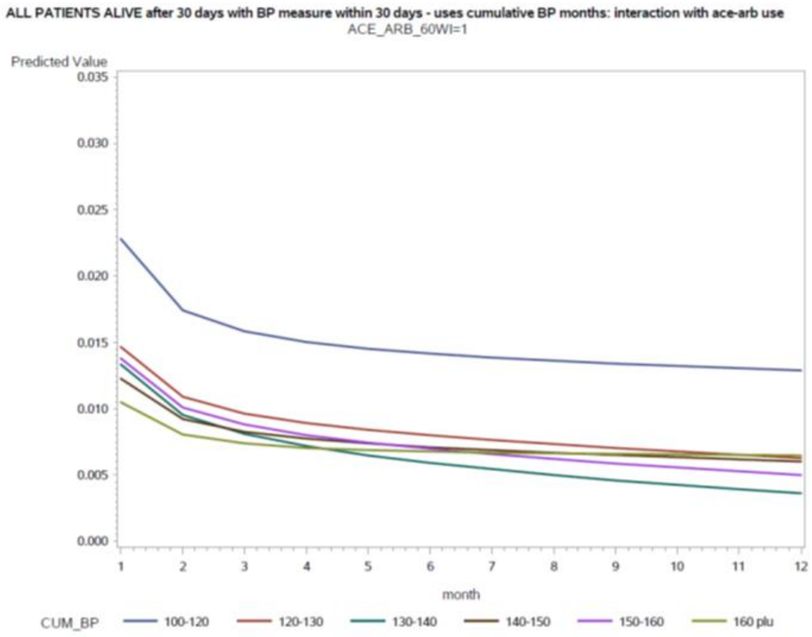

© 2024 Griffin BR et al. *JAMA Network Open.*

eTable 3. In Patients Without CHF at Baseline, Adjusted Hazard Ratios and 99% Confidence Intervals for Mortality and All-Cause Hospital Readmission Over Time, by Blood Pressure Category

			Systolic Blood Pressure (mm Hg)					
			**<120**	**120-129**	**130-139**	**140-149**	**150-159**	**≥160**
**Days From Hospital Discharge**	**Mortality**	**60**	2.02 (1.63-2.51)<br>p < 0.001	1.25 (1.00-1.57)<br>p = 0.01	1.11 (0.88-1.39)<br>p = 0.3	1.09 (0.85-1.41)<br>p = 0.4	1.19 (0.89-1.60)<br>p = 0.1	Reference
		**90**	2.00 (1.62-2.46)<br>p < 0.001	1.21 (0.97-1.50)<br>p = 0.03	1.03 (0.83-1.28)<br>p = 0.7	1.07 (0.84-1.37)<br>p = 0.5	1.13 (0.85-1.49)<br>p = 0.3	
		**120**	1.97 (1.60-2.42)<br>p < 0.001	1.16 (0.94-1.44)<br>p = 0.07	0.96 (0.77-1.19)<br>p = 0.6	1.05 (0.83-1.34)<br>p = 0.6	1.07 (0.81-1.40)<br>p = 0.5	
		**180**	1.92 (1.55-2.36)<br>p < 0.001	1.08 (0.86-1.34)<br>p = 0.4	0.84 (0.67-1.05)<br>p = 0.04	1.02 (0.80-1.30)<br>p = 0.9	0.95 (0.71-1.28)<br>p = 0.7	
		**270**	1.84 (1.46-2.32)<br>p < 0.001	0.96 (0.74-1.24)<br>p = 0.7	0.68 (0.52-0.88)<br>p < 0.001	0.97 (0.72-1.30)<br>p = 0.8	0.81 (0.55-1.19)<br>p = 0.2	
		**365**	1.77 (1.34-2.32)<br>p < 0.001	0.86 (0.63-1.17)<br>p = 0.2	0.55 (0.40-0.76)<br>p < 0.001	0.92 (0.63-1.34)<br>p = 0.6	0.68 (0.41-1.14)<br>p = 0.054	
	**Readmission**	**60**	1.28 (1.15-1.42)<br>p < 0.001	1.14 (1.02-1.27)<br>p = 0.002	1.07 (0.96-1.20)<br>p = 0.09	1.10 (0.98-1.24)<br>p = 0.04	1.15 (1.00-1.31)<br>p = 0.009	Reference
		**90**	1.21 (1.09-1.34)<br>p < 0.001	1.09 (0.98-1.21)<br>p = 0.04	1.02 (0.92-1.13)<br>p = 0.6	1.06 (0.95-1.19)<br>p = 0.2	1.11 (0.98-1.27)<br>p = 0.04	
		**120**	1.14 (1.03-1.27)<br>p = 0.001	1.04 (0.94-1.15)<br>p = 0.3	0.97 (0.87-1.08)<br>p = 0.5	1.03 (0.92-1.15)<br>p = 0.5	1.08 (0.95-1.23)<br>p = 0.1	
		**180**	1.03 (0.92-1.14)<br>p = 0.5	0.95 (0.85-1.06)<br>p = 0.2	0.88 (0.78-0.98)<br>p = 0.002	0.96 (0.85-1.09)<br>p = 0.4	1.01 (0.88-1.17)<br>p = 0.8	
		**270**	0.87 (0.77-0.99)<br>p = 0.007	0.83 (0.72-0.95)<br>p < 0.001	0.75 (0.66-0.86)<br>p < 0.001	0.87 (0.75-1.02)<br>p = 0.02	0.92 (0.76-1.12)<br>p = 0.3	
		**365**	0.74 (0.63-0.87)<br>p < 0.001	0.72 (0.61-0.85)<br>p < 0.001	0.65 (0.55-0.76)<br>p < 0.001	0.79 (0.64-0.96)<br>p = 0.002	0.84 (0.65-1.08)<br>p = 0.08	

* P-value <0.05

Models were adjusted for age, comorbidities (chronic lung disease, congestive heart failure, dementia, malignancy, unexplained weight loss), laboratory values (hematocrit, blood urea nitrogen, bilirubin, albumin), and kidney-specific variables (AKI stage, community-acquired vs in-hospital AKI, proteinuria, baseline creatinine, discharge creatinine)

© 2024 Griffin BR et al. *JAMA Network Open.*

eTable 4. AKI Stage Stratification: Hazard Ratios and 95% Confidence Intervals for Mortality and Readmission Over Time, by Blood Pressure Category

A. Stage 1 AKI

			Systolic Blood Pressure (mm Hg)					
			<120	120-129	130-139	140-149	150-159	≥160
Days From Hospital Discharge	Mortality	60	2.21 (1.81-2.71)<br>p < 0.001	1.38 (1.11-1.70)<br>p < 0.001	1.21 (0.98-1.50)<br>p = 0.02	1.14 (0.90-1.45)<br>p = 0.1	1.22 (0.92-1.61)<br>p = 0.07	Reference
		90	2.17 (1.78-2.64)<br>p < 0.001	1.33 (1.08-1.63)<br>p < 0.001	1.13 (0.92-1.40)<br>p = 0.1	1.11 (0.88-1.40)<br>p = 0.2	1.15 (0.89-1.50)<br>p = 0.2	
		120	2.13 (1.75-2.59)<br>p < 0.001	1.28 (1.04-1.56)<br>p = 0.002	1.06 (0.86-1.30)<br>p = 0.5	1.08 (0.86-1.35)<br>p = 0.4	1.09 (0.84-1.42)<br>p = 0.4	
		180	2.05 (1.68-2.50)<br>p < 0.001	1.19 (0.96-1.46)<br>p = 0.04	0.92 (0.74-1.14)<br>p = 0.3	1.02 (0.80-1.29)<br>p = 0.9	0.98 (0.74-1.30)<br>p = 0.9	
		270	1.94 (1.56-2.40)<br>p < 0.001	1.06 (0.84-1.34)<br>p = 0.5	0.74 (0.58-0.95)<br>p = 0.002	0.93 (0.70-1.24)<br>p = 0.5	0.84 (0.58-1.20)<br>p = 0.2	
		365	1.83 (1.43-2.33)<br>p < 0.001	0.95 (0.71-1.26)<br>p = 0.6	0.60 (0.45-0.82)<br>p < 0.001	0.85 (0.59-1.22)<br>p = 0.2	0.71 (0.44-1.15)<br>p = 0.07	
	Readmission	60	1.33 (1.20-1.47)<br>p < 0.001	1.20 (1.08-1.33)<br>p < 0.001	1.13 (1.01-1.25)<br>p = 0.004	1.17 (1.05-1.32)<br>p < 0.001	1.19 (1.04-1.36)<br>p = 0.001	Reference
		90	1.25 (1.13-1.39)<br>p < 0.001	1.14 (1.03-1.26)<br>p = 0.001	1.07 (0.96-1.19)<br>p = 0.09	1.12 (1.01-1.26)<br>p = 0.007	1.15 (1.01-1.30)<br>p = 0.005	
		120	1.18 (1.07-1.31)<br>p < 0.001	1.08 (0.98-1.20)<br>p = 0.04	1.02 (0.92-1.13)<br>p = 0.7	1.08 (0.96-1.20)<br>p = 0.09	1.11 (0.97-1.26)<br>p = 0.04	
		180	1.05 (0.95-1.17)<br>p = 0.2	0.98 (0.88-1.09)<br>p = 0.7	0.92 (0.83-1.03)<br>p = 0.047	0.99 (0.88-1.12)<br>p = 0.8	1.03 (0.89-1.18)<br>p = 0.6	
		270	0.89 (0.78-1.00)<br>p = 0.01	0.85 (0.74-0.96)<br>p = 0.001	0.79 (0.69-0.90)<br>p < 0.001	0.87 (0.75-1.01)<br>p = 0.02	0.92 (0.76-1.11)<br>p = 0.2	
		365	0.75 (0.64-0.87)<br>p < 0.001	0.73 (0.62-0.86)<br>p < 0.001	0.68 (0.58-0.80)<br>p < 0.001	0.77 (0.63-0.93)<br>p = 0.001	0.82 (0.64-1.06)<br>p = 0.046	

B. Stage 2+3 AKI

			Systolic Blood Pressure (mm Hg)					
			<120	120-129	130-139	140-149	150-159	≥160
Days From Hospital Discharge	Mortality	60	2.20 (1.57-3.07)<br>p < 0.001	1.33 (0.94-1.88)<br>p = 0.04	1.16 (0.81-1.66)<br>p = 0.3	1.00 (0.67-1.49)<br>p = 0.9	1.31 (0.83-2.05)<br>p = 0.13	Reference
		90	2.15 (1.55-2.98)<br>p < 0.001	1.30 (0.92-1.82)<br>p = 0.047	1.07 (0.76-1.52)<br>p = 0.6	0.99 (0.67-1.46)<br>p = 0.9	1.22 (0.79-1.88)<br>p = 0.2	
		120	2.11 (1.53-2.91)<br>p < 0.001	1.27 (0.91-1.77)<br>p = 0.07	0.99 (0.70-1.40)<br>p = 0.9	0.99 (0.68-1.44)<br>p = 0.9	1.14 (0.74-1.76)<br>p = 0.4	
		180	2.03 (1.46-2.81)<br>p < 0.001	1.21 (0.86-1.71)<br>p = 0.2	0.84 (0.59-1.21)<br>p = 0.2	0.98 (0.65-1.45)<br>p = 0.9	1.00 (0.62-1.62)<br>p > 0.99	
		270	1.91 (1.33-2.75)<br>p < 0.001	1.13 (0.75-1.69)<br>p = 0.4	0.66 (0.43-1.03)<br>p = 0.02	0.96 (0.58-1.59)<br>p = 0.8	0.82 (0.43-1.57)<br>p = 0.4	
		365	1.80 (1.17-2.77)<br>p < 0.001	1.05 (0.64-1.73)<br>p = 0.8	0.52 (0.30-0.91)<br>p = 0.002	0.94 (0.49-1.82)<br>p = 0.8	0.67 (0.28-1.59)<br>p = 0.2	
	Readmission	60	1.15 (0.96-1.36)<br>p = 0.04	0.99 (0.83-1.18)<br>p = 0.9	0.93 (0.78-1.11)<br>p = 0.3	1.02 (0.84-1.23)<br>p = 0.8	1.00 (0.80-1.24)<br>p = 0.9	Reference
		90	1.07 (0.90-1.26)<br>p = 0.3	0.94 (0.79-1.11)<br>p = 0.3	0.87 (0.73-1.03)<br>p = 0.03	0.96 (0.80-1.16)<br>p = 0.6	0.96 (0.78-1.19)<br>p = 0.7	
		120	0.99 (0.84-1.17)<br>p = 0.9	0.89 (0.75-1.05)<br>p = 0.07	0.81 (0.68-0.96)<br>p = 0.001	0.91 (0.75-1.09)<br>p = 0.2	0.93 (0.75-1.16)<br>p = 0.4	
		180	0.86 (0.72-1.03)<br>p = 0.03	0.79 (0.66-0.95)<br>p = 0.001	0.70 (0.59-0.85)<br>p < 0.001	0.81 (0.66-1.00)<br>p = 0.009	0.87 (0.68-1.12)<br>p = 0.2	
		270	0.69 (0.55-0.87)<br>p < 0.001	0.67 (0.53-0.85)<br>p < 0.001	0.57 (0.46-0.72)<br>p < 0.001	0.68 (0.52-0.90)<br>p < 0.001	0.79 (0.56-1.12)<br>p = 0.08	
		365	0.56 (0.42-0.75)<br>p < 0.001	0.57 (0.42-0.77)<br>p < 0.001	0.47 (0.35-0.63)<br>p < 0.001	0.58 (0.40-0.83)<br>p < 0.001	0.72 (0.45-1.14)<br>p = 0.06	

Models were adjusted for age, comorbidities (chronic lung disease, congestive heart failure, dementia, malignancy, unexplained weight loss), laboratory values (hematocrit, blood urea nitrogen, bilirubin, albumin), and kidney-specific variables (AKI stage, community-acquired vs in-hospital AKI, proteinuria, baseline creatinine, discharge creatinine).

© 2024 Griffin BR et al. *JAMA Network Open.*
