# Supplementary material for: Blood Pressure, Readmission, and Mortality Among Patients Hospitalized With Acute Kidney Injury
Source: JAMA Netw Open. 2024 May 13;7(5):e2410824. doi: 10.1001/jamanetworkopen.2024.10824 (PMC11091759; doi:10.1001/jamanetworkopen.2024.10824)
Supplement: Supplement 2. — Data Sharing Statement [file jamanetwopen-e2410824-s002.pdf]

Data Sharing Statement

Griffin. Blood Pressure, Readmission, and Mortality Among Patients Hospitalized With Acute Kidney Injury. *JAMA Netw Open*. Published May 13, 2024.
doi:10.1001/jamanetworkopen.2024.10824

Data
Data available: No

Additional Information
Explanation for why data not available: The data that support the findings of this study are available from the US Department of Veterans Affairs (VA). The VA data are made freely available to researchers behind the VA firewall with an approved VA study protocol. For more information, please visit https://www.virec.research.va.gov or contact the VA Information Resource Center (VIReC) at VIReC@va.gov.
